# Items

**Published:** 1885-12

**Authors:** 


### ITEMS.

A NEW JOURNAL.—A prospectus has been issued, announcing the advent of a new journal, to be published by G. P. Putnam & Sons, of New York, and to be devoted, especially, to the discussion of crime, pauperism, insanity, and idiocy, and to the care of the defective and criminal classes. Although these interests are of great magnitude, so far no special journal seems to have been devoted, exclusively, to the consideration of them.

This one is to be under the editorial management of F. H. Wines, Secretary of *The Illinois State Board of Charities*, who has had large experience in this work. The first number is to appear next month.

